# Disparities in healthcare-seeking behaviors and associated costs between Venezuelan migrants and Colombians residing in Colombia

**DOI:** 10.1186/s12939-024-02289-y

**Published:** 2024-10-07

**Authors:** Priya Agarwal-Harding, Brielle Ruscitti, Donald S. Shepard, Arturo Harker Roa, Diana M. Bowser

**Affiliations:** 1 William F. Connell School of Nursing, Boston College, 140 Commonwealth Avenue, Chestnut Hill, MA 02457 USA; 2 The Heller School of Social Policy and Management, Brandeis University, 415 South Street, Waltham, MA 02453 USA; 3 School of Government Alberto Lleras Camargo, Universidad de los Andes, Carrera 1° N° 19-27, Bloque AU, piso 2, Bogotá, Colombia

**Keywords:** Health access, Health behaviors, Health cost, Health system, Health equity, Venezuelan migrants, Integration

## Abstract

**Background:**

Colombia, which hosts over 3 million of the Venezuelan diaspora, is lauded for its progressive approach to social integration, including providing migrants access to its universal health coverage system. However, barriers to healthcare persist for both migrant and host populations, with poorly understood disparities in healthcare-seeking behaviors and associated costs. This is the first study to link healthcare-seeking behaviors with costs for Venezuelan migrants in Colombia, encompassing costs of missing work or usual activities due to healthcare events.

**Methods:**

We use self-reported survey data from Venezuelan migrants and Colombians living in Colombia (September-November 2020) to compare healthcare-seeking behaviors and cost variables by nationality using two-sampled t-tests or Chi-square tests (*X*^*2*^). The International Classification of Diseases was used to compare reported household illnesses for both populations. Average health service direct costs were estimated using the Colombian Government’s *Suficiencia* database and self-reported out-of-pocket (OOP) payments for laboratory and pharmacy services. Indirect costs were calculated by multiplying self-reported days of missed work or usual activities with estimated income levels, derived by matching characteristics using the *Gran Enquesta Integrada de Hogares* database. We calculate economic burdens for both populations, combining self-reported healthcare-seeking behaviors and estimated healthcare service unit costs across six healthcare-seeking behavior categories.

**Results:**

Despite similar disease profiles, Venezuelan migrants are 21.3% more likely to forego formal care than Colombians, with 746.3% more Venezuelans reporting lack of health insurance as their primary reason. Venezuelan women and uninsured report the greatest difficulties in accessing health services, with accessing medications becoming more difficult for Venezuelan women during the COVID-19 pandemic. Colombians cost the health system more per treated illness event (US$40) than Venezuelans (US$26) in our sample, over a thirty-day period. Venezuelans incur higher costs for emergency department visits (123.5% more) and laboratory/ pharmacy OOP payments (24.7% more).

**Conclusions:**

While Colombians and Venezuelans share similar disease burdens, significant differences exist in access, cost, and health-seeking behaviors. Increasing Venezuelan health insurance enrollment and tackling accessibility barriers are crucial for ensuring healthcare equity and effectively integrating the migrant population. Findings suggest that improving migrant access to primary healthcare would produce savings in Colombian healthcare expenditures.

**Supplementary Information:**

The online version contains supplementary material available at 10.1186/s12939-024-02289-y.

## Background

Border violence, poverty, and food insecurity in Venezuela have forced over 6 million Venezuelans to leave the country, the majority of whom travel through or settle in the neighboring country of Colombia [1, 2]. This makes Venezuelans one of the largest populations of displaced persons globally, with 28–39% currently residing in Colombia [3–5].

Despite funding challenges, Colombia has garnered international praise for its welcoming approach to the influx of migrants in recent years, including integrating migrants into its existing social schemes [6–8]. Other South American countries, such as Peru, Brazil, and Chile, have also received large numbers of Venezuelan migrants and have ranging integration and social support efforts [9, 10]. Although barriers to effective access to healthcare remain across countries, Colombia’s approach has generally been regarded as progressive and a positive model for other countries with large displaced populations [4, 9, 11].

However, the growing number of Venezuelan migrants has put a strain on the Colombian health system [8, 12, 13]. In addition to the increased utilization of the health system, many Venezuelans arrive with existing medical conditions and high medical needs associated with their migration status, the humanitarian crisis, and the weakened health system in Venezuela [14–16]. These inputs have had important impacts on healthcare system costs in Colombia, with costs of care for the migrant population being estimated at approximately 16 billion pesos per year [15]. Additionally, the pressure of the influx of migrants, together with an economic recession in Colombia and the lingering impacts of COVID-19, have also increased reliance on social services and subsidies for the Colombian host population, which includes large populations of internally displaced individuals, indigenous groups, and other vulnerable communities [7, 17–19]. This has provided a further stressor to the system and, in some cases, created tensions between the host and migrant populations [20].

However, research remains limited on approaches to integrate migrants into health systems and ensure healthy equity, both in terms of the impacts on health systems themselves as well as on the experiences and health outcomes for both host and migrant populations [21–23]. A key challenge to generating this information has been that data collecting information on migrant experiences is scarce, as migration-related questions are commonly left out of regularly administered surveys [8, 9, 18, 24]. As countries grapple with various approaches to integrating migrants into their health systems, including extending Universal Health Coverage to these populations, as in Colombia, additional analysis of the costs associated with integrating migrants into health systems is also urgently needed [8, 25].

### Integration of Venezuelan migrants in the Colombian health system

Despite changes to Colombia’s political and socio-economic contexts since the early 2010s, the country has remained committed to integration and providing access to basic health services for Venezuelan migrants entering the country [7, 26]. Notably, the Special Permit of Permanence (*Permiso Especial de Permanencia* – PEP) provides temporary residency to Venezuelan migrants for up to two years and access to Colombia’s Universal Health Coverage System—the General System of Social Security in Health (GSSSH). Most recently and in response to the continued migrant crisis and the COVID-19 pandemic, the Colombian government implemented the *Estatuto Temporal de Protección para Migrantes Venezolanos* (ETPMV) in February 2021, which extended the temporary permit for residence for Venezuelan migrants from the previously allowed two years to ten years and made this the new legal pathway for Venezuelan migrants to gain residency in Colombia. Replacing the PEP, the ETPMV similarly provides access to formal employment, public education, and enrollment into the GSSSH [4, 27].

The implementation of the GSSSH has improved financing, access, and utilization of services for Colombians and the extension of GSSSH access to Venezuelan migrants shows significant promise for attaining similar benefits for the migrant population. While disparities in benefits still exist for those within the bottom income quintile [28–30], the GSSSH has extended insurance to over 80% of the Colombian population, increased access to free services by 42% for low-income populations, and increased utilization of ambulatory care by approximately 20% for rural residents [31]. This provision of near universal health coverage through the GSSSH has corresponded with steady increases in general government expenditure on health (% total health expenditures) from 55% in 1995 to 73% in 2020 despite fluctuations due to political unrest and economic uncertainty [34, 35].

However, while having access to the same GSSSH system as Colombians, Venezuelan migrants remain the largest uninsured population in Colombia, with only approximately 26% of individuals having insurance through either the contributory or subsidized schemes as of 2021, compared to 98% of Colombians [4, 9]. Colombians have a 25-fold higher enrollment rate in contributory insurance and are therefore more likely to utilize consultations compared with Venezuelans [19]. Though recent evidence suggests that the gaps in insurance enrollment between Colombians and Venezuelans have narrowed since the implementation of the ETPMV, large disparities in enrollment are still present [32]. A key barrier to enrollment in the GSSSH regimes (contributory or subsidized regimes) is the requirement of legal residency status through the PEP, ETPMV, or other legal pathway, including proper documentation and the completion of several administrative steps [4, 8, 19, 33]. Those without the PEP or legal resident status and who are consequently unenrolled from any GSSSH regime have access to very limited services through emergency care through a publicly financed safety net funded through general taxation [29, 34]. Venezuelan migrants living in Colombia are also more likely to work in the informal sector [15], and fall within lower income quintiles, with household incomes, on average 17% lower than the Colombian host population [35]. These vulnerabilities increase the likelihood of Venezuelan migrants’ impoverishment due to seeking health services [9].

### Healthcare-seeking behaviors and healthcare costs

Globally and within Colombia, numerous barriers exist to seeking healthcare for migrant populations, including discrimination and negative attitudes toward migrants, lack of information about available services and necessary authorization, economic factors, and fear of deportation due to legal status [9, 15, 24, 36–40]. The additional stress of COVID-19 on both systems and individuals exacerbated these barriers and existing vulnerabilities, including those related to access to health services, housing insecurity, loss of employment, and food insecurity, to name a few [41, 42]. In many cases, these barriers have resulted in disparities in both health outcomes and access to healthcare services between migrant and host communities [38].

While it is generally acknowledged that Venezuelan migrants have severe and unique health needs [15], few analyses have been carried out that examine Venezuelan migrant healthcare-seeking behaviors and how these might differ from the Colombian host population [43]. Analyses that use self-reported data that can capture behaviors across both inpatient and outpatient providers, in contrast to hospital billing data, are especially limited [43]. In this same vein, there is a lack of literature estimating and comparing indirect costs associated with care for migrant and host populations and few studies have been able to characterize the economic burdens of care for the two populations [13].

A recent study carried out by Médecins sans Frontières found that only 67% of Venezuelan migrants who needed care sought care, primarily due to a lack of information, fears of discrimination, and lack of insurance [18]. However, this analysis was limited to two Colombian municipalities of La Guajira and Norte de Santander, which border Venezuela, and did not include comparisons with the Colombian host population. A report by Profamilia also examined Venezuelan migrant utilization of select health services in six cities with high migratory flows, finding that between 2018 and 2019, there was a 46% average increased demand for health services [15]. This report also did not include comparisons with the Colombian host populations, nor implications for health costs. However, other studies have shown that Venezuelan migrants’ rates of uninsurance have also been found to impact facility costs. Using hospital billing data, Prada et al. [13] examined health resource use and associated costs for Venezuelan patients with and without insurance. The authors found that Venezuelan migrants without insurance and therefore covered by the municipal government or hospital social support system as part of the publicly funded safety net, as described above, cost 2.37 times more than those insured by the GSSSH in Colombia. Another recent study by Zambrano-Barragan et al. [44], examining how COVID-19 impacted access to health services for Venezuelan migrants in Colombia, highlighted that economic factors, including low wages and indirect costs associated with missing work, were barriers to seeking care. These losses in wages had significant impacts not only on the individual who sought care, but also on the subsistence of large family groups to which they belonged [44]. This evidence highlights the importance of further assessing how these direct and indirect costs may contribute to the overall costs of seeking care for both Venezuelans and Colombians across facilities and settings, toward providing equitable healthcare for all.

Using the context of Colombia and self-reported survey data collected from 5,159 Venezuelan migrants and 2,971 Colombians living in Colombia from September to November 2020, this paper examines the self-reported healthcare-seeking behaviors of Venezuelan migrants and Colombians and the impact of these behaviors on health costs, both to patients and to the health system. The findings of this paper aim to provide important information on the ongoing integration efforts for Venezuelan migrants in Colombia by more accurately representing the impact of the migrant situation on migrants’ and Colombian host populations’ experiences with the health system and their associated economic burden. To the authors’ knowledge, this is the first study to link healthcare-seeking behaviors with costs for Venezuelan migrants in Colombia. The results presented in this paper also answer a call by prior researchers to examine innovative health system approaches for responding to growing epidemiological and demographic demands on their populations, including pathways to health equity and integration in countries grappling with large migrant populations [45].

## Methods

### Data collection

Telephone surveys (*n* = 8,130) were conducted with Venezuelan migrants and Colombians residing in the 60 municipalities with the highest population of Venezuelan migrants in Colombia. Together, these municipalities host approximately 82% of the Venezuelan population living in Colombia. Telephone surveys were carried out during the first wave of the COVID-19 pandemic, from September through November 2020, and prior to the ETPMV announcement and COVID-19 vaccination campaign by the Colombian government, which both took place in February of 2021. In each municipality, we determined the necessary sample to be approximately 70 Venezuelans and 50 Colombians per municipality in the telephone survey to achieve sufficient 95% confidence intervals for differences between the populations on individual questions. This sampling calculation, a sufficient sample size to understand the conditional probability of utilizing health services by insurance status, guaranteed a large enough sample for this observational study [4]. The survey was conducted by an experienced Colombian survey research firm.

The survey captured information from both Colombian citizens and Venezuelan migrants on demographics, health insurance status, work/economic activity, health behaviors, and access to and payment of healthcare services. Insurance status captured the health insurance plan that each respondent was enrolled in (contributory or subsidized) or if the respondent was uninsured (not affiliated with any formal insurance scheme).

The telephone survey was developed in English and then translated to Spanish. The content of the survey was validated and adapted to the local context by a team of experts who made up the study consortium. Surveys were piloted with respondents who were not part of the final sample. The final survey comprised 68 mostly multiple-choice questions and took an average of 25 min to complete.

The broad sampling frame was all low-income Colombians and Venezuelan migrants living in Colombia. In order to arrive at an appropriate sample for the study, we used purposive and snowball sampling methods to recruit survey respondents. Initial volunteers were recruited from diverse settings, and in partnership with local NGOs working with migrant populations in Colombia. Initial respondents (seeds) were randomly selected and were asked at the end of a completed interview to refer another eligible respondent. For effective recruitment, each respondent was given mobile airtime compensation of 3 USD for their own response, plus 1 USD for a referred contact’s response until the target sample was reached in each municipality.

The inclusion criteria for participation in the survey ensured comparability between Colombian and Venezuelan respondents. The inclusion criteria for Colombian citizens included: being a Colombian national; falling within the lowest two urban socio-economic household classifications (*estrato)*; and being at least 18 years of age. *Estratos* are calculated by the Colombian government based on houses’ exterior physical characteristics and conditions of the surrounding areas, with a total of six groups ranging from least (Estrato 6) to most vulnerable (Estrato 1) and entitle households to receive subsidies on public utilities (water, sanitation, electricity, and gas). Approximately 90% of the Colombian population fall within the lowest three *estratos* [46]. Inclusion criteria for Venezuelan migrants included: being a Venezuelan national; falling within the lowest three *estratos*; and being at least 18 years of age. Estrato 3 was included for Venezuelan migrants as it was deemed necessary to account for the fact that many migrants, despite being classified in a higher socio-economic classification, may actually experience lower socio-economic status after discounting classification by housing situation, given that migrants are more likely to reside in multi-family homes with shared expenses that may resemble homes with larger incomes and housing expenses.

### Analysis

Descriptive statistics were calculated for healthcare utilization and cost variables by nationality (Venezuelan and Colombian) and the overall survey population using two-sampled t-tests or Chi-square tests (*X*^*2*^). Differences between and within populations by self-reported insurance status and sex (female and male) were also calculated.

The International Classification of Diseases (ICD) version 10, in place during the same time period as the survey, was utilized to classify the open-ended responses reporting illness experiences for respondents or other members of their households within the past 30 days (*N* = 116), for both Venezuelan migrants and Colombians [47]. The ICD serves as a standardized and systematic classification system for the interpretation of disease data [48, 49]. Classified responses were integrated with survey questions for self-reported illnesses experienced.

The self-reported healthcare-seeking behavior patterns for both Venezuelan migrants and Colombians were combined with healthcare service unit costs to calculate the economic burden across six healthcare-seeking behavior categories: emergency department visits, hospital visits, public sector consultations, private sector/specialist services, pharmacy and labs, and indirect costs. Each category of costs is reported separately for Colombians and Venezuelans and the total costs are reported as the sum of all individual costs for both populations. As the survey did not differentiate between emergency department, hospital visits, and outpatient consultations within public sector healthcare services, we used data from the Colombian Government’s *Suficiencia* database to estimate the proportion of visits utilized in each of these facility types as well as the average direct cost for a health visit in each category. The *Suficiencia* includes data on the number of services provided to both Colombian and Venezuelan patients enrolled in any health scheme within Colombia and the costs associated with these visits by calendar day of utilization. Data was collected from a single day—January 5, 2020—around the same time as our survey, including a total of 128,592 patient visits to health facilities (2,092 visits by Venezuelan migrants).

Costs for consultations, pharmacy, laboratory testing, and medical supplies were included in total costs for each healthcare-seeking behavior. Costs for additional out-of-pocket costs were estimated using self-reported out-of-pocket payments spent on pharmacy and laboratory testing collected by the telephone survey. These costs were used to calculate the total costs for self-reported hospital visits, public sector consultations, and emergency department visits combined with the level of health facility visited. Similarly, *Suficiencia* data was used to estimate average costs for private sector or specialist consultations for both population groups, including any services provided at private sector facilities. In cases where individuals reported visiting multiple facilities (*N* = 3), costs for visiting all facilities were included in the analysis. Reported dental visits were excluded from analyses on healthcare-seeking behaviors and costs, as dental care derives from different healthcare needs. Visits to Red Cross health clinics and self-medication or home remedies were also excluded from the cost analysis, as they either incurred no facility-level cost or fell outside the Colombian social security system.

Indirect costs were defined as the monetary cost of days missed at work or other usual activities due to a healthcare event (including visiting a healthcare facility). The number of missed days was then multiplied by each individual’s calculated income level. Individual incomes were estimated and extracted from the *Gran Enquesta Integrada de Hogares* (GEIH) database from The National Administrative Department of Statistics (DANE) in Colombia [50]. Average daily incomes were estimated for individuals by dividing annual incomes by 244 total working days in Colombia [51] and merged with survey data by grouping individuals based on the following variables: geographic department, socioeconomic strata, age range, sex, urban or rural residence, and formal or informal employment. Informal employment was defined as an individual who is employed but does not receive a pension or social security payments, according to the DANE definition. Likewise, formal sector employment was defined as an individual who is employed and receives both types of benefits [52]. Municipalities surveyed were categorized as rural or urban using data from the 2005 Colombian census through DANE, including variables for access to public works and services, and population density [50] and in consultation with experts in Colombia. This classification followed the definition of urban and rural areas utilized by DANE [53]. All costs were calculated in US$2020 to match the timing of the survey data collection, using the average rate for the year ($1 USD= $3,692.77 COP) [54]. Costs were inflated to 2024 USD and reported in the Annex [55].

## Results

Table 1 describes the characteristics of the overall survey population by nationality. Venezuelan migrants make up 63.4% of the sample and Colombians make up 36.5%. Colombian respondents are slightly older than Venezuelans (mean of 37.6 vs. 32.7, *p* < 0.001). Venezuelans reside in slightly larger households than Colombians (mean of 4.9 vs. 4.7; *p* < 0.01). The proportion of female to male respondents is about equal in both populations (62.3% for Venezuelans and 61.2% for Colombians). Slightly more Venezuelans than Colombians report being employed at the time of the survey (52.3% compared with 43.1%, *p* < 0.01), although a significantly larger percent of Colombians are employed in the formal sector (19.3% compared with 7.4%, *p* < 0.001). Significantly more Venezuelans than Colombians also report having a university degree or higher (12.8% vs. 5.0%, *p* < 0.001). Mean daily incomes, estimated using the GEIH, show that Colombians earn slightly more than Venezuelans (*p* < 0.05), with Colombians earning about US$0.84 equivalent per day and Venezuelans earning approximately US$0.82 equivalent per day.

**Table 1:** Summary of survey respondent characteristics, by Colombians and Venezuelans (%, N)

		Venezuelan (*N* = 5,159)	Colombian (*N* = 2,971)	*p*-values
Total respondents		63.4%	36.5%	—
Respondents per municipality (Standard Deviation)		86.0 (13.4)	49.5 (3.9)	—
Age in years (Standard Deviation)		32.7 (10.1)	37.6 (13.8)	< 0.001
Household size (Standard Deviation)		4.9 (2.5)	4.7 (2.4)	0.001
Female		62.3%	61.2%	
Employed		52.3%	43.1%	0.005
Employed in formal sector		7.4%	19.3%	< 0.001
Completed University		12.8%	5.0%	< 0.001
Affiliated with any insurance		25.5%	96.5%	< 0.001
Mean daily income (Standard Deviation)		$0.82 ($0.36)	$0.84 ($0.37)	0.014
Reported illness event in household (last 30 days)		15.9%	14.4%	0.070
Affiliated with any insurance		24.8%	97.0%	< 0.001
Took any action for illness event		72.4%	77.5%	0.263
Did not seek formal healthcare		41.5%	34.2%	0.001
Stopped work due to illness event		60.1%	56.9%	0.431
Days stopped work (Standard Deviation)		8.5 (8.2)	9.5 (9.2)	0.149

**Note** Percents in non-indented rows are shown out of total survey respondents by Venezuelans and Colombians, respectively. Percents in indented rows are shown out of those who reported an illness event in their household (*N* = 819 Venezuelans, *N* = 427 Colombians). Numbers in brackets are standard deviations for the mean values. Mean daily income was calculated from the GEIH, using a currency conversion rate of $1 USD= $3,692.77 COP (average 2020 rate), to capture rates at the time of the study [54]. Statistical significance levels are shown to test the mean values between Venezuelans and Colombians.

Of the 8,130 individuals included in the survey, 15.3% (1,246; not reported in Table 1) report experiencing any illness (medical or dental) within their household within the last 30 days, with slightly more Venezuelan households experiencing illness compared to Colombians (15.9% vs. 14.4%). There are no significant differences in rates of reported household illness by nationality. However, significant differences are present within groups by insurance status (*p* < 0.001). Of the 16% of Venezuelans who reported a household illness in the past 30 days (*N* = 819), 75.2% are unaffiliated with any insurance scheme and only 24.8% had insurance. This was compared to 97.0% of Colombians who report a household illness and had some type of insurance coverage (subsidized or contributory) (*N* = 414) versus only 3.0% that are uninsured. While significant differences are not found between Colombians and Venezuelans for likelihood to take any action for a reported illness (77.5% vs. 72.4%, respectively), Venezuelans are significantly less likely than Colombians to seek any form of formal healthcare for a reported illness (41.5% vs. 34.2%, *p* < 0.05).

A breakdown of self-reported illnesses experienced for Venezuelans and Colombians is included in the Annex (A1), however, differences between the two groups are minimal. The majority of Colombians and Venezuelans (60%, *N* = 749) report seeking care due to an acute illness (48%, *N* = 600) or an accident (12%, *N* = 155), with only 2% (*N* = 20) reporting that they sought care due to a regular health visit. Dental problems also constitute a substantial share of reported illnesses (24%, *N* = 304).

For Colombians and Venezuelans that provided open-ended responses for the self-reported illnesses experienced (*N* = 116), the leading types of illness, grouped using ICD-10 categories, are also similar for both population groups. The leading illnesses self-reported by both Venezuelans (*N* = 70) and Colombians (*N* = 46) are diseases of the respiratory system, pregnancy, childbirth, and the puerperium, and diseases of the digestive system (see Annex A2 and A3 for full results of the open-ended responses).

**Fig. 1:**
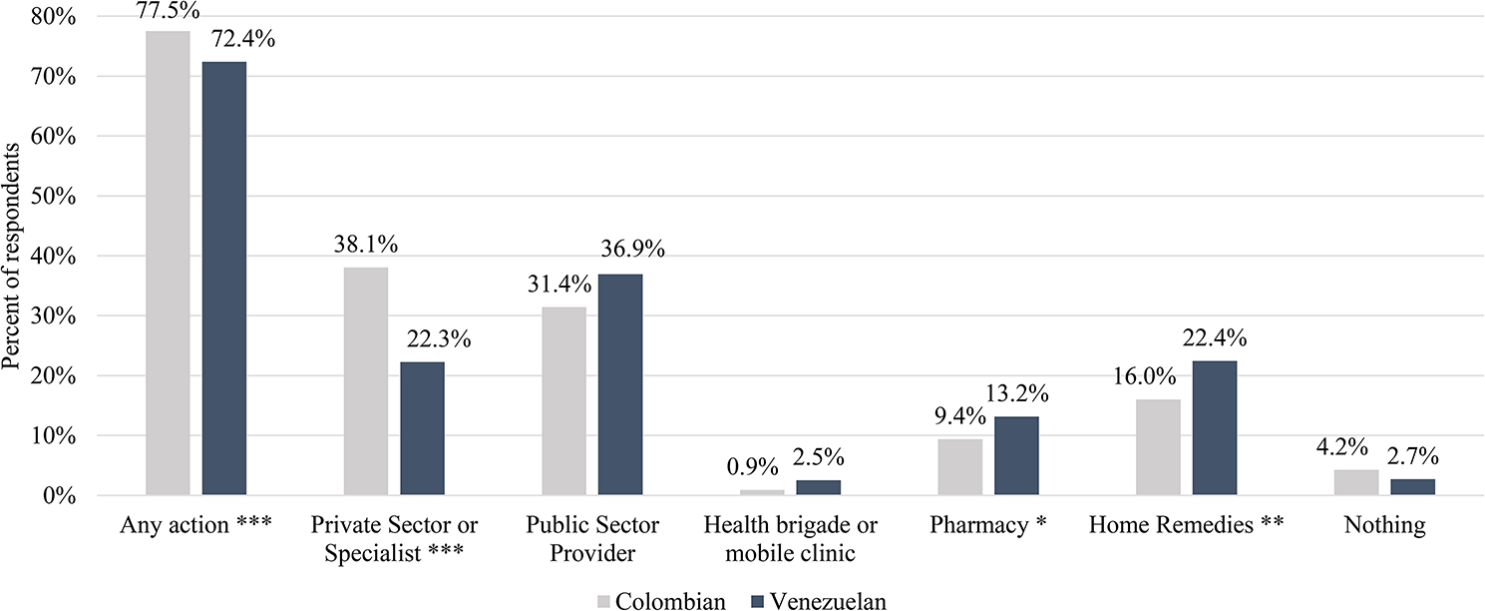
Healthcare-seeking behaviors by nationality (%) *N* = 924. **Note** Percent totals are shown for both populations out of the total number of individuals who reported taking any action for their reported household illness (*N* = 924, *N* = 331 Colombians and *N* = 593 Venezuelans), except for the “any action” bars, which display the total number of respondents who took any action for a health issue, as a percentage of those who reported a household illness (*N* = 1,246, *N* = 427 Colombians and *N* = 819 Venezuelans). Visits to public sector providers include all visits to public hospitals, emergency departments, and clinics, including telehealth consultations. Those who sought care for dental services were excluded from this analysis. Statistical significance levels between Colombians and Venezuelans are shown at: *p* < 0.001 (***), *p* < 0.01 (**), and *p* < 0.05 (*)

However, despite these similar disease profiles, Venezuelan migrants and Colombians report different care-seeking behaviors and barriers to care. Figure 1 shows significant differences between Venezuelans and Colombians in terms of the actions taken for a reported household illness, including visits to private sector providers or specialists and use of home remedies. Colombians are significantly more likely to report visiting a private sector provider or specialist (38.1% vs. 22.3%, *p* < 0.001) than Venezuelan migrants. In contrast, Venezuelans are more likely than Colombians to report using home remedies (22.4% vs. 16.0%, *p* < 0.05), as well as using any public sector facility (36.9% vs. 31.4%), visiting a health brigade/mobile clinic (2.5% vs. 0.9%), or visiting a pharmacy (13.2% vs. 9.4%), although these were not found to be statistically significant.

**Fig. 2:**
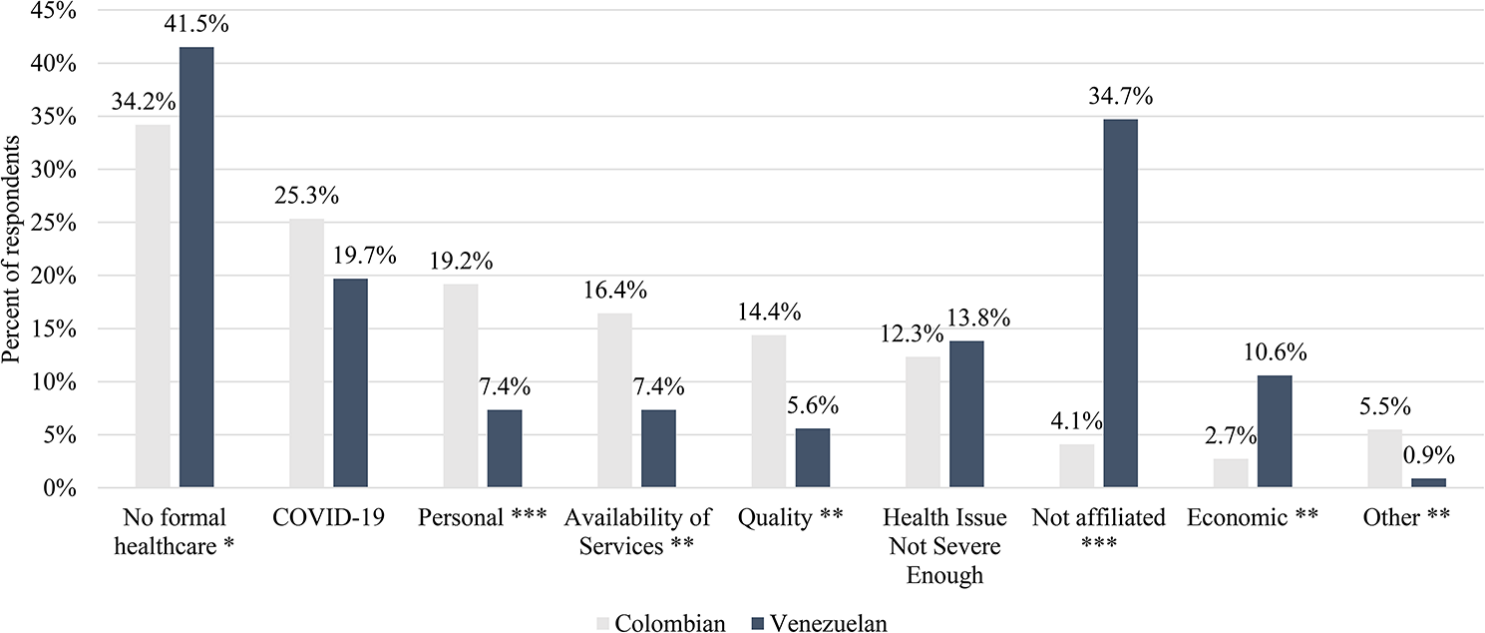
Primary Reason for not seeking formal healthcare services, *N* = 486. **Note** Percent totals are shown for both populations out of the total number of respondents who reported not seeking any type of formal healthcare for their reported household illness (*N* = 486, *N* = 146 Colombians and *N* = 340 Venezuelans), except for the “no formal healthcare” bar, which displays the total number of respondents who did not use formal healthcare as a proportion of the total number of respondents who reported a household illness within the past 30 days. Statistical significance levels between Colombians and Venezuelans are shown at: *p* < 0.001 (***), *p* < 0.01 (**), and *p* < 0.05 (*)

The greatest differences between Venezuelans and Colombians are found in terms of reasons for not seeking formal healthcare services. Figure 2 shows that among those who reported not seeking any type of formal healthcare service for a health or dental issue (*N* = 486), Venezuelan migrants are 21.3% more likely to report not seeking any type of formal care than Colombians (41.5% and 34.2%, respectively; *p* < 0.005) with 746.3% more Venezuelans reporting that lack of affiliation with the health system was their primary reason for not seeking formal care (34.7% and 4.1%, respectively; *p* < 0.001). Venezuelans are also 292.6% more likely to cite economic reasons (10.6% vs. 2.7%; *p* < 0.01) and 12.2% more likely to cite that the health issue is not severe enough (13.8% vs. 12.3%) as reasons for not seeking care than Colombians. Colombians are more likely to report not seeking formal care primarily due to personal reasons (159.5% more; *p* < 0.001), concerns over a lack of health services available (121.6% more; *p* < 0.01), concerns over the quality of care that they would receive (157.1% more; *p* < 0.01), or other reasons (143.8% more; *p* < 0.01). Additionally, while fear of contracting COVID-19 is a common concern between both Colombians and Venezuelan migrants, 28.4% more Colombians report this as a primary reason for not seeking care.

**Fig. 3:**
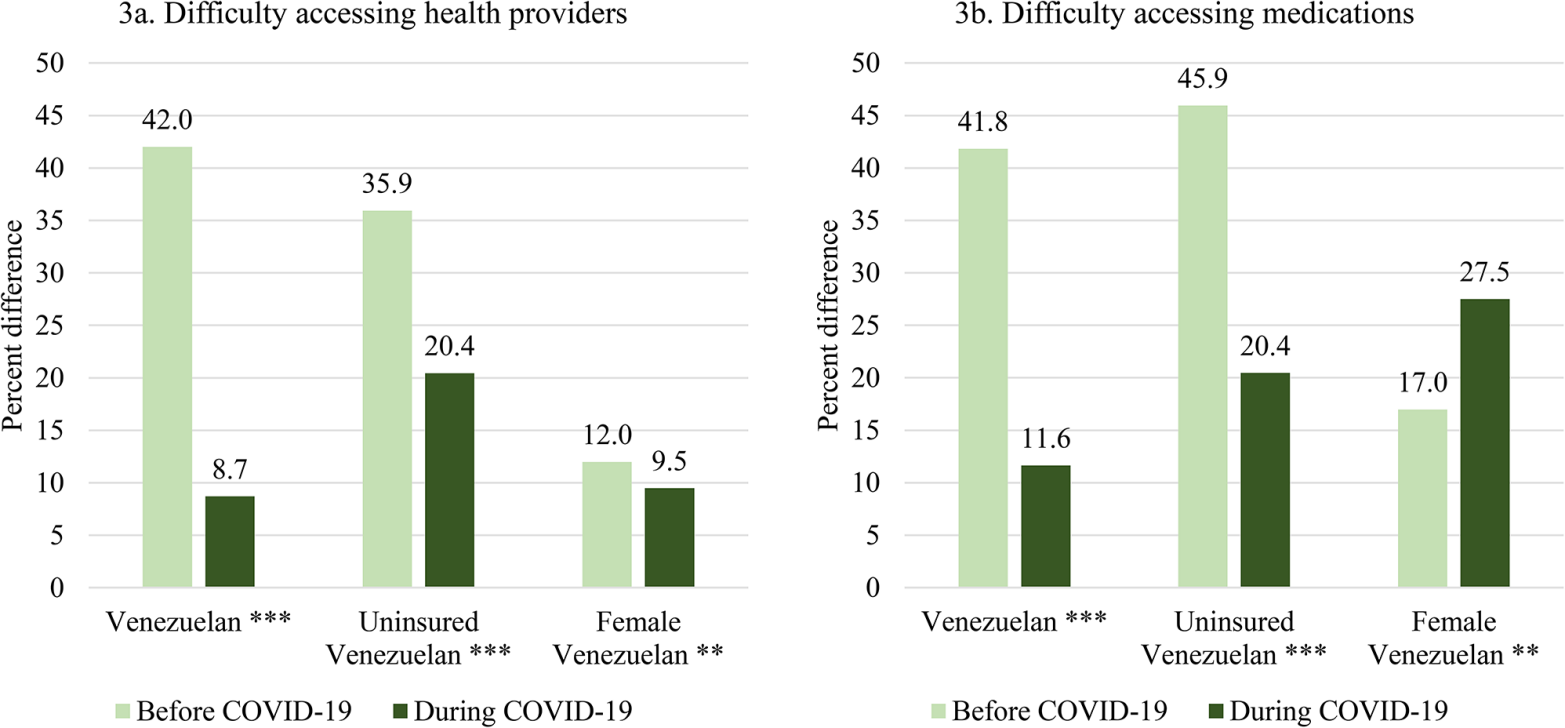
Percent difference in difficulty accessing health services before vs. during COVID-19 for Venezuelans and by sub-population. **Note** Percent differences are displayed out of the total number of individuals who reported difficulties in accessing health providers (*N* = 3,567; *N* = 1,554 Colombians and *N* = 2,013 Venezuelans) and medications (*N* = 3,971; *N* = 1,659 Colombians and *N* = 2,312 Venezuelans). Percent differences are reported for Venezuelans compared with Colombians and subpopulations of Venezuelans by uninsured vs. insured (*N* = 869 and *N* = 769 uninsured individuals who reported difficulties in accessing health providers and medications, respectively) and female vs. male (*N* = 1,004 and *N* = 833 females who reported difficulties in accessing health providers and medications, respectively). Labels display the baseline group for comparison

Figure 3a and 3b show that Venezuelans report having significantly greater difficulty in accessing health services and medications both before and during the COVID-19 pandemic, compared to Colombians (*p* < 0.001). The first two bars on panels 3a and 3b show that prior to the pandemic, 42.0% more Venezuelans compared to Colombians report difficulties in accessing healthcare providers and 41.8% more report difficulties in accessing medications, respectively. While this disparity narrowed during the pandemic, Venezuelans still report higher rates of difficulty in accessing medications (11.6% more) and healthcare providers (8.7% more) than Colombians.

Figure 3a and 3b also show that Venezuelan uninsured and Venezuelan women, in comparison to Venezuelan insured and Venezuelan men, are more likely to have trouble accessing health services both before and during the pandemic and this became even more difficult for female Venezuelans during the pandemic. The middle two bars on panels 3a and 3b show that prior to the pandemic, 35.9% more uninsured Venezuelans compared to insured Venezuelans report difficulties in accessing healthcare providers and 45.9% more face difficulties accessing medications. While this disparity also narrowed during the pandemic, uninsured Venezuelans still report higher rates of difficulty in accessing healthcare providers and medications (20.4% more for both) during the pandemic in comparison to insured Venezuelans.

As the final bars in Fig. 3b show, for female Venezuelans, it became even more difficult to access medications in comparison to male Venezuelans during the pandemic, increasing from a 17.0% higher difference prior to the pandemic (*p* < 0.01) to a 27.5% difference during the pandemic (*p* < 0.001).

Both before and during the pandemic, Venezuelan uninsured and women face the highest difficulties in accessing medications and are among the groups with the highest difficulty in accessing health providers (see Annex A4 for full results). Disparities between Colombian insured and uninsured individuals and between Colombian men and women are not statistically significant (see Annex A5 for results).

Table 2 displays the estimated economic burden for five healthcare-seeking behaviors and the indirect costs associated with those behaviors for surveyed respondents. As described previously, Colombians and Venezuelans report similar disease burdens within their households over the thirty days prior to the survey, with 11.4% of Colombian households reporting a non-dental illness event and 11.6% of Venezuelan households. Colombians cost more per treated illness event treated than Venezuelans, with Colombians costing approximately US$40 per illness event and Venezuelans costing approximately US$26 per illness event. When examining costs for each type of healthcare-seeking behavior, the costs of care are higher for Colombian households as they seek more hospital visits (51.0% more; 9.1% vs. 5.4%), private sector providers and specialists (14.8% more; 26.1% vs. 22.5%), and public sector consultations (189.7% more; 3.8% vs. 0.1%) than Venezuelan households. However, Venezuelan households incur a larger share of both emergency department costs (123.5% more; 13.1% vs. 3.1%) and out-of-pocket payments for laboratory and pharmacy services (24.7% more; 32.3% vs. 25.2%), resulting in a higher overall share of direct costs (as a percent of total costs) for services utilized (8.5% more, 73.4% vs. 67.4%). Colombian households incur a larger share of indirect costs associated with missing work or usual activities due to a healthcare event for their household’s most recent illness episode (20.3% more; 32.6% vs. 26.6%).

**Table 2:** Estimated economic burden for healthcare-seeking behaviors reported over 30 days prior to survey (US$ 2020)

	Colombian					Venezuelan				
Total sample	13,939					25,160				
Survey respondents	2,971					5,159				
Illness events in sample	340					601				
Percent of households with an illness event	11.4%					11.6%				
Total economic costs for illness events treated ($US)	13,463					15,829				
Economic cost per event treated ($US)	39.60					26.34				
	**N**	**(%)**	**Unit cost** **(US$)**	**Total cost** **(US$)**	**Total cost (%)**	**N**	**(%)**	**Unit cost** **(US$)**	**Total cost** **(US$)**	**Total cost** **(%)**
Direct health sector costs ***	418	(122.8%)	78.33	9,072.39	67.4%	724	(120.5%)	42.94	11,620.62	73.4%
Hospital visits	24	(7.0%)	51.36	1,230.80	9.1%	30	(5.0%)	28.68	856.71	5.4%
Emergency Department	26	(7.7%)	15.93	419.29	3.1%	180	(29.9%)	11.54	2,071.14	13.1%
Private sector or specialist	126	(37.1%)	27.88	3,513.07	26.1%	132	(22.0%)	27.02	3,566.69	22.5%
Public sector consultation	46	(13.6%)	11.03	510.44	3.8%	6	(1.0%)	2.72	16.49	0.1%
Pharmacy & labs OOP costs *	195	(57.4%)	17.43	3,398.78	25.2%	377	(62.7%)	13.55	5,109.60	32.3%
Indirect healthcare costs **	223	(65.6%)	19.69	4,391.05	32.6%	390	(64.9%)	10.79	4,208.08	26.6%

**Note** Direct and indirect costs are displayed in US$ equivalent, using a conversion rate of $1 USD = $3,692.77 Colombian pesos (average 2020 rate) [54]. The Annex (A6) includes costs equivalent to January 1, 2024 rates (1 USD= $3,887.50 COP) [55]. Economic costs per event treated were derived by dividing estimated total costs by the total sample of individuals who reported that their household had experienced an illness event in the 30 days prior to the survey. Ns reflect the number of respondents reporting each healthcare-seeking behavior within their household, with percents in brackets indicating the proportion of respondent households using each behavior out of the total number of illness events reported. Totals do not add up to the total number of illness events in the sample as this includes the number of respondent households who incurred out-of-pocket expenditures in addition to the number of respondent households who visited health facilities and excludes dental visits and other services outside the Colombian social security system. Unit costs were taken from the *Suficiencia* for visits to hospitals, emergency departments, private sector or specialists, and public sector consultations. Average unit costs for pharmacy and laboratory OOP payments and indirect costs were estimated using the telephone survey. Total direct costs are the sum of unit costs for public sector visits (excluding private sector or specialist and OOP costs). Total costs by healthcare-seeking behavior were derived by multiplying the Ns by the unit costs for each service. Percentages of total costs reflect direct and indirect costs as a proportion of total economic costs for illness events treated in formal facilities. Statistical significance levels between total costs for Colombians and Venezuelans are shown at: *p* < 0.001 (***), *p* < 0.01 (**), and *p* < 0.05 (*). The significance level for direct health sector costs includes visits to hospitals, emergency departments, private sector or specialists, and public sector consultations.

## Discussion

This is one of the first studies to use self-reported health-seeking behaviors to understand access to and costs of healthcare for Venezuelan migrants in Colombia. The results are important to not only understand the burden on the healthcare system in Colombia, but also patterns and inequities in access to healthcare services for many migrant populations in countries around the globe. Findings presented above show that while the Venezuelan migrant and the Colombian host population exhibit similar disease burdens, access, health-seeking behaviors, and costs differ significantly between the two populations. The results of this study provide useful evidence to healthcare providers and policymakers in Colombia as well as in other healthcare systems with large number of non-citizens and uninsured.

Although complete and detailed information was not available on specific diseases that individuals faced, the comparison of self-reported illnesses experienced in the 30 days prior to the survey and classification of open-ended responses by ICD-10 categories revealed similar burdens of disease. Leading digestive and respiratory diseases for both population groups are consistent with symptoms of COVID-19 and may reflect an increased incidence of COVID-19 among both population groups at the time of the survey in November 2020. The fact that both groups reported conditions related to pregnancy, childbirth, and the puerperium also seem to accurately reflect the persistent nature of these conditions and match findings from other scholars that COVID-19 may have increased the rate of pregnancy-related complications, including maternal deaths, ruptured ectopic pregnancies, and maternal depression [19, 56].

The differing patterns in health-seeking behavior for Venezuelans versus Colombians presented above suggest that access to health insurance is a critical factor for equalizing access to health services between migrant and host populations. More migrants reported seeking care through self-directed means, including home remedies and pharmacies, as well as through public sector providers and health brigades. By contrast, Colombians were significantly more likely to visit private sector providers or specialists. These preferences were also reflected in reasons given for not seeking formal healthcare services, where Venezuelans were less likely to report seeking formal healthcare due to issues of accessibility, including lack of insurance affiliation, economic reasons, or delays in seeking care due to the severity of the condition. These patterns are most likely related to the fact that many Venezuelan migrants did not have access to formal health insurance at the time of the survey, with approximately 73.6% of Venezuelan telephone survey respondents remaining uninsured (compared to just 3.6% of Colombians) [4], though our analysis did not control for municipality differences, such as with fixed effects, which may have impacted significance levels.

However, recent government policies in Colombia show promise to further integrate the Venezuelan migrant population and increase equitable access to healthcare services. As previously described, in an effort to address the continued migrant crisis in Colombia and the additional hurdles of COVID-19, the Colombian government implemented the ETPMV in February 2021. This policy has the potential to grant approximately 1.8 million Venezuelan migrants who were residing in Colombia prior to the policy’s announcement access to formal employment and public services, including enrollment to the GSSSH [4, 27]. With evidence that enrollment into a health insurance regime can equalize access to healthcare services for migrants and host populations [4], this policy shows promise for further facilitating access to healthcare for many Venezuelans.

There is some evidence presented above that although disparities in healthcare access narrowed slightly over the COVID-19 pandemic, accessing healthcare services is still quite difficult for Venezuelans in comparison to Colombians, and especially for uninsured and female Venezuelans as compared to insured and male Venezuelans. This pattern was especially noted for access to medications. Studies of migrant populations in numerous settings have found that uninsured and female migrants face additional difficulties in accessing healthcare services, as additionally vulnerable populations [24, 57–61]. These additional challenges frequently stem from the financial burdens of care, transportation difficulties, child-care responsibilities, and issues of acceptability of care, to name a few. Furthermore, migrant women are often disproportionally impacted by disasters, such as COVID-19, due to a number of vulnerability factors faced by this population [62]. Within Colombia, Doocy et al. [12] found that migrants in Norte Santander also faced critical challenges in accessing non-emergent care, including accessing a majority of medications, which are not covered by the government’s safety-net system. While Doocy et al.’s study did not examine differences in access by gender, it can be expected that the barriers, as described above, as well as COVID-19 travel restrictions may have compounded to make accessing medications especially challenging for female Venezuelan respondents in our study.

An important contribution of this paper is linking self-reported healthcare-seeking behaviors with associated healthcare costs, including estimated indirect costs of care. This information is critical for understanding where health inequities lie and the true costs of care for Venezuelan migrant and Colombian host populations residing within Colombia, both for the health system itself and for individuals. The results above show that Venezuelan migrants incur lower costs per illness event in our survey sample and had lower rates of utilization for most formal healthcare services except for emergency department visits and out-of-pocket payments for pharmaceuticals and laboratory services. This finding is consistent with evidence from the US and other countries around the world that have found that based on migrants’ patterns of lower utilization of non-emergent healthcare services, younger age distributions, and other factors, such as the healthy migrant effect, migrants are generally a smaller financial burden on their health systems than the host population [24, 63–65]. This finding is also contrary to the common assumption that Venezuelan migrants are a cost burden on the Colombian health system and is especially important as emergency visits cost approximately 123.7% times higher than a normal public sector consultation for Venezuelans ($11.03 and $2.72, respectively, see Table 2). However, it is important to note that the analysis presented above only includes a single illness event per household and does not include multiple illnesses that individuals may have experienced within households. With our survey carried out during the COVID-19 pandemic, it is likely that more than one individual per household may have experienced an illness event requiring care at formal health facilities, and that the costs reported in this paper may therefore be underestimated for both populations. We were also unable to factor in specific diagnoses and lengths of hospital stays into the analysis, which would likely be large cost drivers [66].

The variation in the distribution of total costs of care between Venezuelans and Colombians is driven in part by lower average unit costs per healthcare visit for Venezuelans compared to Colombians according to *Suficiencia* data utilized, especially for public sector consultations, hospital visits, and emergency department visits. This is likely due to Venezuelans utilizing emergency department visits for non-emergent health conditions that could be treated through primary care settings and driven by barriers to primary care, such as lack of insurance and the expectation of high out-of-pocket costs for services [12, 67]. This pattern of non-emergent care through emergency departments has also been found in many other settings where migrants or other vulnerable groups lack access to insurance and face barriers in accessing preventative care [68–72]. Along with these barriers to preventative care, it may also be likely that Venezuelans who do utilize public sector consultations and hospitals for their healthcare needs may receive lower levels of service quality than their Colombian peers, as high uninsurance rates result in a lack of reimbursement for services provided [67, 73]. Last, it is important to note that data on average unit costs of services were obtained for a single day—January 5, 2020—which was a Sunday, and Venezuelan migrants made up only about 1.6% of total health facility visits (2,092 visits). Future studies would benefit from using a larger database that may more accurately represent unit costs of services, if available, as well as further investigate the drivers of health-seeking behaviors and the associated economic burden, as described above.

Enrolling more Venezuelan migrants into a health insurance regime, as the government is aiming to do with the recent ETPMV, can narrow disparities in health service utilization and promote the utilization of primary and preventative health services over emergent care. As our findings show, if more Venezuelans were able to access primary care services and non-emergent emergency department visits were reduced, such as through enrollment in the GSSSH, it is likely that savings in healthcare expenditures would be produced. With Venezuelans exhibiting lower levels of utilization for most formal healthcare services, as described above, improving access to preventative healthcare and increasing healthcare utilization among migrants would be unlikely to financially overburden the Colombian health system. These cost savings would be further bolstered by the longer-term cost savings produced by expanding access to primary and preventative healthcare services, which is supported by a large body of evidence [74–77]. To evaluate this causal relationship, future studies may wish to examine the impacts of the ETMPV policy change on the disparities between Colombians and Venezuelans examined in this paper and evaluate changes to the associated economic burdens for both populations.

It is important to note several limitations of this study. First, this study was based on self-reported survey data, and as such, the results may be biased from differences in problem recognition and culture between migrant and host communities, or fear of negative stereotyping or legal consequences from response. For this reason, it is recommended that future studies triangulate results over different sources of data, if available. Second, our survey was also limited in terms of the diagnostic and health utilization variables it included, as well as questions on individual income and urban vs. rural residence. This may have impacted our precision in estimating the types of facilities visited by respondents and may have impacted our estimations of income and therefore indirect costs associated with care. However, estimates of income were based on nationally representative figures and widely used classifications by DANE, which gives us more confidence in our estimates. Last, although the survey sample is large, some subgroups, such as uninsured Colombians, comprise smaller samples, which may have limited the power of our statistical results.

## Conclusion

While Colombian host and Venezuelan migrant populations exhibit similar disease burdens and conditions, access, cost, and health-seeking behaviors differ significantly between the populations. Although some disparities in healthcare access narrowed slightly over the COVID-19 pandemic, results show that accessing healthcare services—and especially non-emergent services, such as medications—is still difficult for Venezuelan migrants, and especially Venezuelan uninsured and women. Our estimate of the economic burden associated with these behaviors indicates that if these disparities in accessibility of health services were reduced, such as by enrolling more Venezuelans into a formal insurance regime, savings in healthcare expenditures would be produced. With recent policies, such as the ETPMV, that will facilitate access to health insurance and other social protection schemes to better integrate the migrant population into Colombian society, addressing barriers to care and facilitating more equal access to the Colombian health system will be critical to ensure the success of the policy and address the continued pressures of the migration crisis in the country.

## Electronic supplementary material

Below is the link to the electronic supplementary material.

Supplementary Material 1

## Data Availability

Data available from corresponding author, PA-H, upon reasonable request.

## Abbreviations

COP: Colombian pesos
COVID-19: Coronavirus disease of 2019
DANE: The National Administrative Department of Statistics
ETPMV: Estatuto Temporal de Protección para Migrantes Venezolanos
GEIH: Gran Enquesta Integrada de Hogares
GSSSH: General System of Social Security in Health
ICD-10: The International Classification of Diseases version 10
NGO: Non-governmental organization
OOP: Out-of-pocket payments
PEP: Permiso Especial de Permanencia
R2HC: Research for Health in Humanitarian Crises
UK: United Kingdom
UHC: Universal Health Coverage
US: United States
USD: United States Dollars
