# Supplementary material for: Disparities in healthcare-seeking behaviors and associated costs between Venezuelan migrants and Colombians residing in Colombia
Source: Int J Equity Health. 2024 Oct 7;23:202. doi: 10.1186/s12939-024-02289-y (PMC11460058; doi:10.1186/s12939-024-02289-y)
Supplement: Supplementary file 1 — Supplementary Material 1 [file 12939_2024_2289_MOESM1_ESM.docx]

**Annex**

**A1: Self-reported illness event by nationality**

Note: Percents are shown out of the total number of Colombian and Venezuelan respondents that reported experiencing a specific illness in their household in the past 30 days (N=427 Colombians, N=819 Venezuelans). Answers given to open-ended responses on type of illness experienced were integrated into existing sub-categories when possible. Open-ended responses that did not fit into one of the existing sub-categories are classified as “other reason”.

**A2: Venezuelan Health Issues Faced**

| **ICD 10 Group** | **Frequency (N=70)** | **Percent (%)** |
| --- | --- | --- |
| Disease of the respiratory system | 10 | 14% |
| Symptoms, signs and abnormal findings not elsewhere classified | 7 | 10% |
| Pregnancy, childbirth and the puerperium | 7 | 10% |
| Disease of the digestive system | 7 | 10% |
| Disease of the skin and subcutaneous tissue | 5 | 7% |
| Disease of the musculoskeletal system | 4 | 6% |
| No Category | 4 | 6% |
| Endocrine, nutritional and metabolic diseases | 2 | 3% |
| Diseases of the nervous system | 2 | 3% |
| Disease of the genitourinary system | 2 | 3% |
| Injury poisoning and other consequences of external causes | 2 | 3% |
| Infectious and parasitic disease | 1 | 1% |
| Disease of the eye and adnexa | 1 | 1% |
| Diseases of the ear and mastoid process | 1 | 1% |
| Diseases of the circulatory system | 1 | 1% |
| Congenital malformations, deformations and chromosomal abnormalities | 1 | 1% |
| External causes of morbidity and mortality | 1 | 1% |
| Factors influencing health status and contact with health services | 1 | 1% |
| Neoplasm | 0 | 0% |
| Blood, blood-forming organs, and immune mechanism diseases | 0 | 0% |
| Mental and behavioral disorders | 0 | 0% |
| Conditions originating in the perinatal period | 0 | 0% |

Note: Percents are shown out of the total number of Venezuelan respondents (N=70) that reported experiencing a specific illness in their household in the past 30 days. Open-ended responses on type of illness experienced were classified by ICD-10 illness categories when possible. Symptoms, signs and abnormal findings not elsewhere classified (ICD-10 number 18) captures individual responses describing general illness, not associated with other ICD-10 categories. No category captures individual responses describing interactions with the healthcare system but that did not indicate a specific illness.

**A3: Colombian Health Issues Faced**

| **ICD 10 Grouping** | **Frequency (N=46)** | **Percent (%)** |
| --- | --- | --- |
| Disease of the digestive system | 6 | 13% |
| Disease of the musculoskeletal system | 3 | 7% |
| Pregnancy, childbirth and the puerperium | 4 | 9% |
| Disease of the respiratory system | 5 | 11% |
| Symptoms, signs and abnormal findings not elsewhere classified | 3 | 7% |
| Mental and behavioral disorders | 3 | 7% |
| Diseases of the nervous system | 3 | 7% |
| Injury poisoning and other consequences of external causes | 3 | 7% |
| Diseases of the ear and mastoid process | 2 | 4% |
| Disease of the genitourinary system | 2 | 4% |
| No Category | 1 | 2% |
| Diseases of the circulatory system | 1 | 2% |
| Disease of the eye and adnexa | 0 | 0% |
| Neoplasm | 0 | 0% |
| Infectious and parasitic disease | 0 | 0% |
| Blood, blood-forming organs, and immune mechanism diseases | 0 | 0% |
| Endocrine, nutritional and metabolic diseases | 0 | 0% |
| Disease of the skin and subcutaneous tissue | 0 | 0% |
| Conditions originating in the perinatal period | 0 | 0% |
| Congenital malformations, deformations and chromosomal abnormalities | 0 | 0% |
| External causes of morbidity and mortality | 0 | 0% |
| Factors influencing health status and contact with health services | 0 | 0% |

Note: Percents are shown out of the total number of Colombian respondents (N=46) that reported experiencing a specific illness in their household in the past 30 days. Symptoms, signs and abnormal findings not elsewhere classified (ICD-10 number 18) captures individual responses describing general illness, not associated with other ICD-10 categories. No category captures individual responses describing interactions with the healthcare system but that did not indicate a specific illness.

**A4: Percent experiencing difficulty accessing health services before vs. during COVID-19**

Note: Percent differences are displayed as subpopulations who reported difficulties in accessing health providers (N=3,567, N=1,554 Colombians and N=2,013 Venezuelans) and medications (N=3,971, N=1,659 Colombians and N=2,312 Venezuelans).

**A5: Percent difference in difficulty accessing health services before vs. during COVID-19 for Colombians by sub-population**

Note: Percent differences are displayed out of the total number of Colombians who reported difficulties in accessing health providers (N=1,554 Colombians) and medications (N=1,659 Colombians). Percent differences are reported for uninsured vs. insured and female vs. male. Labels display the baseline group for comparison.

**A6: Estimated economic burden for healthcare-seeking behaviors reported over 30 days prior to survey (US$ 2024)**

|  | **Colombian** | | | | | | | **Venezuelan** | | | | | | | |
| --- | --- | --- | --- | --- | --- | --- | --- | --- | --- | --- | --- | --- | --- | --- | --- |
| Total sample | 13,939 | | | | | | | 25,160 | | | | | | | |
| Survey respondents | 2,971 | | | | | | | 5,159 | | | | | | | |
| Illness events in sample | 340 | | | | | | | 601 | | | | | | | |
| Percent of households with an illness event | 11.4% | | | | | | | 11.6% | | | | | | | |
| Total economic costs for illness events treated ($US) | 12,789 | | | | | | | 15,036 | | | | | | | |
| Economic cost per event treated ($US) | 37.61 | | | | | | | 25.02 | | | | | | | |
|  | **N** | | **(%)** | **Unit cost**  **(US$)** | | **Total cost**  **(US$)** | **Total cost (%)** | **N** | | **(%)** | **Unit cost**  **(US$)** | | **Total cost**  **(US$)** | | **Total cost**  **(%)** |
| **Direct health sector costs ***** | 418 | (122.8%) | | 74.40 | 8,617.94 | | 67.4% | 724 | (120.5%) | | 40.79 | 11,038.53 | | 73.4% | |
| Hospital visits | 24 | (7.0%) | | 48.79 | 1,169.15 | | 9.1% | 30 | (5.0%) | | 27.24 | 813.80 | | 5.4% | |
| Emergency Department | 26 | (7.7%) | | 15.13 | 398.29 | | 3.1% | 180 | (29.9%) | | 10.96 | 1,967.40 | | 13.1% | |
| Private sector or specialist | 126 | (37.1%) | | 26.48 | 3,337.10 | | 26.1% | 132 | (22.0%) | | 25.67 | 3,388.03 | | 22.5% | |
| Public sector consultation | 46 | (13.6%) | | 10.48 | 484.87 | | 3.8% | 6 | (1.0%) | | 2.58 | 15.66 | | 0.1% | |
| Pharmacy & labs OOP costs * | 195 | (57.4%) | | 16.56 | 3,228.53 | | 25.2% | 377 | (62.7%) | | 12.87 | 4,853.65 | | 32.3% | |
| **Indirect healthcare costs **** | 223 | (65.6%) | | 18.70 | 4,171.10 | | 32.6% | 390 | (64.9%) | | 10.25 | 3,997.29 | | 26.6% | |

Note: Direct and indirect costs are displayed in US$ equivalent, using a conversion rate of 1 USD= $3,887.50 COP (54). Economic costs per event treated were derived by dividing estimated total costs by the total sample of individuals who reported that their household had experienced an illness event in the 30 days prior to the survey. Ns reflect the number of respondents who reporting each healthcare-seeking behavior, with percents in brackets indicating the proportion of respondents using each behavior out of the total number of illness events reported. Totals do not add up to the total number of illness events in the sample as this includes the number of respondents who incurred out-of-pocket expenditures in addition to the number of respondents who visited health facilities and excludes dental visits and other services outside the Colombian social security system. Unit costs were taken from the Suficiencia for visits to hospitals, emergency departments, private sector or specialists, and public sector consultations. Average unit costs for pharmacy and laboratory OOP payments and indirect costs were estimated using the telephone survey. Total direct costs are the sum of unit costs for public sector visits (excluding private sector or specialist and OOP costs). Total costs by healthcare-seeking behavior were derived by multiplying the Ns by the unit costs for each service. Percentages of total costs reflect direct and indirect costs as a proportion of total economic costs for illness events treated in formal facilities. Statistical significance levels between total costs for Colombians and Venezuelans are shown at: p<0.001 (***), p<0.01 (**), and p<0.05 (*). The significance level for direct health sector costs includes visits to hospitals, emergency departments, private sector or specialists, and public sector consultations.
